# Primitive Neuroectodermal Tumour (PNET) in subcutaneous abdominal wall: a case report

**DOI:** 10.1186/1477-7800-6-10

**Published:** 2009-03-31

**Authors:** Dibendu Betal, Behnam Shaygi, Ramesh Babu, Kamarul Jamil, Richard J Sainsbury

**Affiliations:** 1 Department of Surgery, St Mary's Hospital, Isle of Wight NHS Trust, Parkhurst Road, Newport, Isle of Wight, PO30 5TG, UK; 2 Department of Histopathology, St Mary's Hospital, Isle of Wight NHS Trust, Parkhurst Road, Newport, Isle of Wight, PO30 5TG, UK

## Abstract

Primitive neuroectodermal tumour (PNET) is a rare tumour mainly found in children under ten years old. It may be broadly categorised into those occurring from the central or peripheral nervous system of which the majority arise centrally. We report a 61 year-old lady who had previous lobular breast cancer presenting with a rapidly expanding lesion in her anterior right upper abdominal wall. Clinically it appeared to be benign, however, histopathology of the excised lesion revealed a localised PNET. This case is an unusual case of a PNET in an adult that is peripheral in nature arising from subcutaneous tissue in the abdominal wall.

## Background

PNETs are rare tumours that are more prevalent in children than adults and occurirng more commonly in the central nervous system than peripheral. Peripheral PNETs are similar to Ewing's sarcoma [1]. We present a case of a peripheral PNET occurring in the subcutaneous abdominal wall.

## Case presentation

A 61 year-old lady presented to clinic with a lesion in her anterior abdominal wall. It was present for four weeks and initially thought to be a sebaceous cyst but had rapidly expanded in that time frame. She had no other symptoms of note. On clinical examination the patient had a lesion measuring 4 × 3 cm firm, fixed, hemispherical with a smooth edge. There was a punctum and some indurated fat around it.

Her past history was of a right-sided breast cancer, she underwent a wide local excision and axillary dissection for a grade 1 lobular carcinoma with no nodal involvement. She underwent adjuvant treatment with radiotherapy and tamoxifen.

She was initially referred due to concern that this lesion was related to her previous breast cancer, however clinically it appeared benign and she underwent elective excision of the lesion under local anaesthesia.

**Figure 1:**
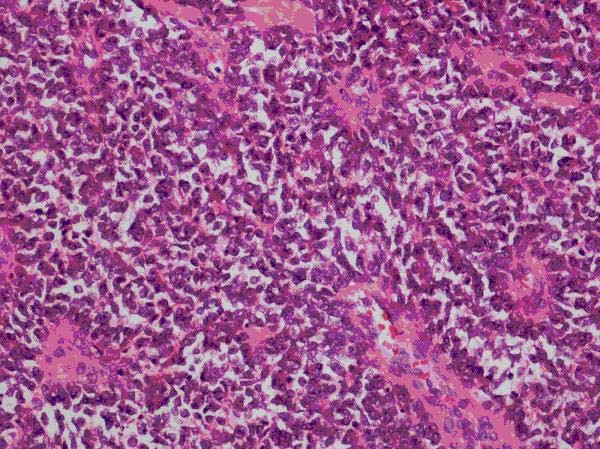
**Rosette formation in cells**.

**Figure 2:**
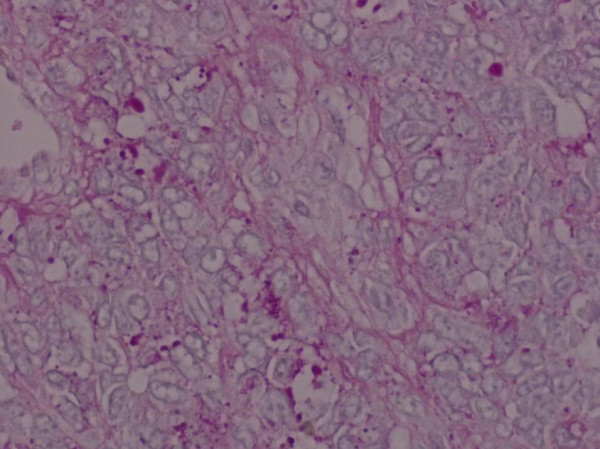
**Glycogen in cells**.

Histopathology revealed a tumour growing as solid sheets diffusely infiltrating the dermis and subcutaneous fat with significant areas of necrosis. The tumour was monomorphic with intermediate sized cells with scanty cytoplasm. There was a hint of rosette formation (Figure 1) but no other differentiating features relating to pigment, gland formation or mucin. Special stains showed cytoplasmic glycogen (Figure 2) and immunohistochemistry showed strong expression of CD99, focal expression of CD56 and focal dot-like expression of cytokeratins, expression of S100 but no expression of other melanoma markers. The combination of morphology and immunochemistry was consistent of the spectrum of primitive neuroectodermal tumours.

She was referred to Oncology for adjuvant therapy. Staging CT scan of the chest, abdomen and pelvis revealed ill defined haziness in the subcutaneous fat of the right upper abdomen but no solid or cystic lesions were apparent. No other significant abnormalities were seen. Whole body bone-scan showed no evidence of bony metastases. Bone marrow trephine biopsy showed normocellular marrow with no evidence of neoplasia.

Although the lesion appeared to be completely excised the recommendation was for adjuvant chemotherapy. She was commenced on 6 cycles of Vincristine, Doxorubicin and Cyclophosphamide.

## Discussion

Primitive neuroectodermal tumours (PNET) are rare tumours that are more prevalent in children. They may be broadly divided into those occurring in the central nervous system such as non pineal supratentorial PNET, medulloblastoma and pineoblastoma. Those occurring peripherally may resemble soft tissue sarcoma (small round blue cell tumours – SRBCT) and are similar to Ewing's sarcoma as they share common chromosomal change. Although Ewing's sarcomas are more common in bone there are examples of extraosseous tumours that may resemble PNET.

There have been a number of case reports for peripheral PNETs including those in the eye [2,3], maxillo-facial [4-6], peripheral limbs [7,8], gynaecological organs [9-11] and intraabdominal [12,13]. There have been reports of skin and subcutaneous PNETs mainly in children, adolescents and young adults [14] and very few reports specifically regarding abdominal subcutaneous PNET and the only ones have been in children [15,16].

On a histopathology level PNETs are highly cellular showing sheets of small round cells with hyperchromatic nuclei. They the progress with neuron-specific enolase expression then onto rosette formation as demonstrated by this case, cytoplasmic glycogen, phenotypic ganglion cell differentiation and finally neurofilament protein expression. Tumours that are undifferentiated by light microscopy, immunochemistry or electron microscopy have been diagnosed as Ewing sarcoma. The majority of Ewing's sarcomas and PNETs have a t(11;22)(q24;q12) translocation, there are smaller cases of t(21;21)(q21,q12) and t(7,22)(q22,12) translocation. [17]

Treatment of PNETs depends on the type of PNET, position in the body, size of the tumour, spread and the age and health of the patient.

## Conclusion

PNETs are a rare form of tumours demonstrating neural differentiation. This case demonstrates an unusual presentation of a PNET in peripheral abdominal subcutaneous tissue in an adult. Treatment involves resection, chemotherapy with or without radiotherapy.

## Competing interests

The authors declare that they have no competing interests.

## Authors' contributions

DB Literature searches, writing the paper. BS Performed surgical procedure. RB Performed surgical procedure. KJ Examined surgical specimen, wrote pathological report and provided histological photographic slides. JRS Supervised final revision of the article

## Consent

Written informed consent was obtained from the patient for publication of this case report and any accompanying images. A copy of the written consent is available for review by the Editor-in-Chief of this journal.
